# A novel breast cancer image classification model based on multiscale texture feature analysis and dynamic learning

**DOI:** 10.1038/s41598-024-57891-5

**Published:** 2024-03-27

**Authors:** Jia Guo, Hao Yuan, Binghua Shi, Xiaofeng Zheng, Ziteng Zhang, Hongyan Li, Yuji Sato

**Affiliations:** 1 Hubei Key Laboratory of Digital Finance Innovation, Hubei University of Economics, Wuhan, 430205 Hubei China; 2 Xiangzhou District People’s Hospital of Xiangyang, Xiangyang, 441100 Hubei China; 3 Faculty of Computer and Information Sciences, Hosei University, Tokyo, 102-8160 Japan; 4 School of Information Engineering, Hubei University of Economics, Wuhan, 430205 Hubei China; 5 Hubei Internet Finance Information Engineering Technology Research Center, Hubei University of Economics, Wuhan, 430205, Hubei China

**Keywords:** Breast cancer image classification, Multi-scale texture analysis, Dynamic learning strategy, Breast cancer, Displays

## Abstract

Assistive medical image classifiers can greatly reduce the workload of medical personnel. However, traditional machine learning methods require large amounts of well-labeled data and long learning times to solve medical image classification problems, which can lead to high training costs and poor applicability. To address this problem, a novel unsupervised breast cancer image classification model based on multiscale texture analysis and a dynamic learning strategy for mammograms is proposed in this paper. First, a gray-level cooccurrence matrix and Tamura coarseness are used to transfer images to multiscale texture feature vectors. Then, an unsupervised dynamic learning mechanism is used to classify these vectors. In the simulation experiments with a resolution of 40 pixels, the accuracy, precision, F1-score and AUC of the proposed method reach 91.500%, 92.780%, 91.370%, and 91.500%, respectively. The experimental results show that the proposed method can provide an effective reference for breast cancer diagnosis.

## Introduction

Cancer, as a serious disease, endangers people’s lives, and its effects are very severe worldwide. In particular, breast cancer is a malignant tumor of the epithelial tissue that seriously threatens women’s lives and health care systems, with a high prevalence rate and a long treatment process^1^. For women, the occurrence rate of breast cancer is only lower than that of lung cancer. Currently, breast cancer has become an essential public health issue of global concern. Early detection and therapy are key to improving the survival rate of breast cancer patients. With the development of medicine, the analysis of histopathological images has become the most widely used method for breast cancer diagnosis. This method is used to diagnose pathology through biological tissue sections. Thus, the nature and type of tumor can be identified, providing an important foundation for a surgeon’s next diagnosis.

Since a rigorous and accurate diagnosis is the core of the treatment of cancer, the analysis of histopathological images has become a hot topic in current research. Pathology-based image analysis and artificial intelligence-assisted diagnosis are playing increasingly significant roles in this field and are critical factors in helping physicians enhance the accuracy of cancer diagnosis^2^. Deep learning in artificial intelligence^3^ has also shown great potential in tumor region identification, tumor microenvironment characterization, prognosis prediction, and so on.

Based on numerous studies, deep learning has been applied to mineral mapping^4^, hyperspectral image classification problems^5^^6^, oral cancer image classification^7^, breast cancer image classification^8^, skin cancer prediction^9^, potato and rice disease prediction^10^, and many others. Among them, in the field of diagnosing breast cancer, histopathological image analysis is an important technique in its early stages, but its efficiency is not guaranteed in practical applications^11^. The poor accuracy of image edge detection^12^, time-consuming and tedious annotation delineation of images^11^, expensive data tagging^13^, and unbalanced dataset selection^14^ have seriously hindered the development of effective computerized diagnostic methods. Among the many technologies that address related issues, convolutional neural networks and recurrent neural networks^15^ in deep learning have shown excellent performance in various vision tasks^16^. However, in some cases, traditional neural networks still have some drawbacks. When the network depth is increased excessively, problems such as accuracy degradation and gradient disappearance may arise^17^. In the application of practical scenarios, detection and interpretation errors may occur, leading to incorrect diagnoses^18^. Thus, many researchers have made strong efforts in the field of histopathological image analysis. In 2014, Xu^19^ proposed a comprehensive framework based on weakly supervised learning combined with machine learning to effectively separate cancerous tissues from digital images and classify them into different types. Researchers have made improvements to convolutional neural networks. In 2018, Zhong^20^ proposed an end-to-end spectral-spatial residual network (SSRN), which improves the precision of deep learning. Zhu^21^ proposed a generative adversarial network (GAN), which is very competitive in the face of complex tasks and highly competitive in the face of complex and nonlinear data analysis tasks.

Combining convolutional neural networks in machine learning with feature extraction algorithms can be far more efficient than traditional manual feature extraction algorithms. Mei^22^ proposed an unsupervised spatial-spectral feature learning strategy that uses a 3-dimensional (3D) convolutional autoencoder (CAE) to maximize the exploration of spatial-spectral structure information and used it for CNN learning. Li^23^ microsupervised the mining of contrast patterns between normal and malignant images using a fully convolutional autoencoder to learn the main structural patterns in normal image patches, making the output diagnostic solution easier to understand. Wang^24^ used adversarial networks in deep learning for sample generation, proposed the Caps-TripleGAN framework, and applied it to hyperspectral image classification. Hameed^25^ implemented the automatic classification of complex cancer cell images by using large amounts of data for training deep learning models. By combining the merits of convolutional and recurrent neural networks. Yan^26^ applied a neural network to breast cancer histopathology image analysis, using a CNN to extract richer multilevel information from case images and then fusing plaque features using an RNN to derive image classification finally.

However, most feature extraction algorithms need to process a large amount of information with many redundant features. In 2020, Feng^27^ proposed a deep manifold preserving autoencoder, which learns discriminative features directly from unlabelled data and then fine-tunes a neural network with labeled training data, preserving the structure of the input dataset from the perspective of popular learning and minimizes the reconstruction error of a large amount of unlabelled data from the perspective of deep learning, thus learning discriminative features. Given the complexity of label relationships in images, graph representations may be difficult to distinguish. Nguyen^28^ proposed the modular graph transformer network (MGTN), which partitions a computational graph into multiple modular-based subgraphs through a preprocessing procedure, and on the basis of the MGTN, different subgraphs can be better propagated in terms of information. By combining a contrast learning method and a transformer model. Hu^29^ proposed a novel unsupervised framework that can effectively extract hyperspectral image features without supervision. By comparing traditional machine learning and deep learning experiments. Boumaraf^30^ concluded that deep learning has better explanations for clinical image classification. Dai^31^ combined the advantages of CNNs and transformers to propose TransMed for multimodal medical image classification, which is able to capture long-distance dependencies between different modalities and shows great potential in the field of medical research. Wang^32^ combined the advantages of convolutional neural networks and capsule networks and proposed histopathological image classification of breast cancer based on deep feature fusion and augmented routing by extracting convolutional features and capsule features simultaneously through a novel two-channel structure. Thilagaraj^33^ combined an artificial fish swarm algorithm with a deep convolutional neural network and proposed the implementation of an improved DCNN for the classification of breast cancer images using an artificial fish swarm model, in which the training data of the deep convolutional neural network are directly provided by the artificial fish swarm algorithm. Hong^34^ proposed the spectral former from the perspective of the order of transformers, which overcomes the limitation of traditional convolutional neural networks by their inherent network backbone. Liu^35^ proposed a hierarchical learning algorithm based on a Bayesian neural network classifier, which builds a visual confusion label tree from the output of a convolutional neural network model, constructs a hierarchical structure for a large number of categories in an image dataset, and automatically determines the hierarchical learning task. In addition, a backtracking algorithm was introduced into the algorithm for reclassification as a way to correct samples that were misclassified during the former classification process. Also, novel evolutionary methods^36–38^ can be used in complex optimization problems.

The main contributions of this paper are listed as follows:

1. Texture feature parameter extraction (TFPE) is used to transform figures into a matrix.
2. An unsupervised dynamic learning mechanism (UDLM) is proposed to classify the matrix generated from TFPE. The UDLM does not require human annotation or pre-parameter learning and achieves unsupervised classification. Moreover, its adaptive evolution capability enables the UDLM to be quickly applied to the classification of different data.

The rest of this paper is organized as follows. “Materials and methods” section introduces the proposed method, “Experiments and results” section introduces the experimental methods and results, and “Conclusion” section presents the discussion.

## Materials and methods

### Texture feature parameter extraction based on a GLCM

A novel breast cancer image classification model (BCICM) is proposed in this section. Image texture information is one of the important bases to reflect whether a breast cancer image is complex. Referring to the related results in the field of image complexity analysis, textural features can be obtained using the grey-level cooccurrence matrix (GLCM) methods. By computing the GLCM of an image to obtain its feature information, Haralick proposed 14 statistical feature parameters, including energy, entropy, contrast, homogeneity, correlation, and variance. Combining the above methods results in 20 feature parameters describing the complexity of a breast cancer image from different aspects, but there is an overlap problem and redundancy among these feature parameters. Therefore, in this paper, four textural parameters with a low correlation that are easy to calculate, including the energy, contrast, inverse difference moment, and correlation, are selected for the complexity perception of a breast cancer image. Assume an $M \times N$ breast cancer image *I* with $N_g$ grey grades. $\left( x_{1}, y_{1}\right)$ and $\left( x_{2}, y_{2}\right)$ are two pixel points in image *I* with distance *d* in the direction of $\theta$. Then, the GLCM of this breast cancer image is calculated by Eq. (1).

1 $$\begin{aligned} \begin{aligned} P(i, j, d, \theta )=\#\{\left( x_{1}, y_{1}\right) ,\left( x_{2}, y_{2}\right) \in M \times N \mid I\left( x_{1}, y_{1}\right) =i, I\left( x_{2}, y_{2}\right) =j \} \end{aligned} \end{aligned}$$

where $\#$ denotes the number of elements in the set. $i,j=0,1,2,..., N_g-1$ represents the grey levels of the two pixels.

The energy (*ASM*) is used to describe the uniformity of the distribution of the breast cancer image. When the elements are concentrated near the diagonal of the GLCM, a smaller *ASM* value indicates that the greyscale distribution is more uniform and the texture is finer; conversely, it indicates that the greyscale distribution is uneven and the texture is rougher. The ASM is calculated by Eq. (2).

2 $$\begin{aligned} A S M=\sum _{i=0}^{N_{g}-1} \sum _{j=0}^{N_{g}-1} P(i, j, d, \theta )^{2} \end{aligned}$$

The contrast (*CON*) is used to reflect the depth of image texture grooves and clarity. In a particular breast cancer image, a clearer image texture means a larger *CON* value, and the opposite means a smaller value. The (*CON*) is calculated by Eq. (3).

3 $$\begin{aligned} C O N=\sum _{i=0}^{N_{g}-1} \sum _{j=0}^{N_{g}-1}(i-j)^{2} P(i, j, d, \theta ) \end{aligned}$$

The inverse difference moment (*IDM*) is a statistical feature parameter that reflects the local texture of a breast cancer image. When the *IDM* value is large, the textures of different regions in the breast cancer image are more homogeneous. The (*IDM*) is calculated by Eq. (4).

4 $$\begin{aligned} I D M=\sum _{i=0}^{N_{g}-1} \sum _{j=0}^{N_{g}-1} \frac{P(i, j, d, \theta )}{1+(i-j)^{2}} \end{aligned}$$

The correlation (*COR*) is used to measure the similarity of the GLCM elements in the row or column direction. When the row or column similarity is high, the *COR* value is larger, and the complexity of the scene is smaller. The opposite also holds. The (*COR*) is calculated by Eq. (5).

5 $$\begin{aligned} C O R=\sum _{i=0}^{N_{g}-1} \sum _{j=0}^{N_{g}-1}\left( i-\mu _{1}\right) \left( j-\mu _{2}\right) / \delta _{1} \delta _{2} \end{aligned}$$

where $\mu _{1}$ and $\mu _{2}$ denote the mean values of the elements along the normalized GLCM in the row and column directions, respectively. $\delta _{1}$ and $\delta _{2}$ represent their mean squared values, respectively.

Therefore, for any breast cancer image, we extract four parameters separately and combine them into a texture feature vector. Details are shown in Eq. (6).

6 $$\begin{aligned} {\textbf {E}}=[A S M, C O N, I D M, C O R] \end{aligned}$$

### Unsupervised dynamic learning mechanism for data classification

Breast cancer-related image data have a wide variety and are difficult to label. Traditional machine learning methods require a large amount of labeled data in the training process, which also leads to slow and expensive training. Based on these factors, a novel unsupervised dynamic learning mechanism (UDLM) for data classification is proposed in this section. Compared with traditional classification methods, the UDLM does not require pretraining or human labeling, and it can efficiently and accurately classify data according to their characteristics.

To evaluate the performance of the classification algorithm, the sum distance is defined by Eq. (7):

7 $$\begin{aligned} SumDis(p_i) & = \sum \limits _{i = 1}^n {Dis(p+i)} \\ Dis(p+i) & = \min (Euc({p_i},center1),Euc({p_i},center2))\end{aligned}$$

where *Euc*(*a*, *b*) is a function used to calculate the Euclidean distance between elements *a* and *b*. From this, it can be seen that each element is divided into the cluster centers closer to it, and the distance between them is calculated into the sum distance. Now, the whole problem is transformed into finding two suitable clustering centers that minimize the sum of distances.

To achieve unsupervised classification, a metaheuristic algorithm is used in the UDLM, and a dynamic learning strategy is added to enhance the accuracy of the algorithm. As a metaheuristic algorithm, the historical optimal position (*pbest*) of each particle and the population optimal position (*gbest*) of all particles are recorded for subsequent calculations. Specifically, a particle with no mass and volume, only coordinates, is used as the basic search unit. Each particle has two sets of coordinates, one representing the center of the first class and the other representing the center of the second class. Each particle corresponds to a value that is calculated by Eq. (7). First, all particles are scattered randomly in the search space. As the search proceeds, particles are clustered towards the optimal particles until the end of the iteration. In each iteration, one particle coevolves with a random neighbor particle. To describe this process more accurately, we assume that of the two particles selected, the one with the smaller sum distance is particle *j* and the one with the larger sum distance is particle *i*. The next positions of particle *i* and particle *j* are calculated by Eq. (8):

8 $$\begin{aligned} \alpha & = (p_i^t + p_j^t)/2\\ \beta &= abs(p_i^t - p_j^t)\\ \chi & = (p_j^t + gbes{t^t})/2\\ \delta & = abs(p_j^t - gbes{t^t})\\ candidate\_p_i^(t+1) &= Gauss(\alpha ,\beta )\\ candidate\_p_j^(t+1) &= Gauss(\chi ,\delta ) \end{aligned}$$

where $p_i^t$ is the personal best position of particle *i* in the *t*th generation, $p_j^t$ is the personal best position of particle *j* in the *t*th generation, $gbes{t^t}$ is the best position of all particles in the *t*th generation, *abs*(*a*, *b*) is a function that is used to calculate the absolute value between *a* and *b*; *Gauss*(*a*, *b*) is a function that is used to calculate the Gaussian distribution with a mean of *a* and a standard deviation of *b*, $candidate\_p_i^{t+1}$ is the candidate position of particle *i* in the $(t+1)$th generation, and $candidate\_p_j^{t+1}$ is the candidate position of particle *j* in the $(t+1)$th generation. To completely describe the evolution of all particles, the pseudo-code of the UDLM is shown in Algorithm 1.

**Algorithm 1:**
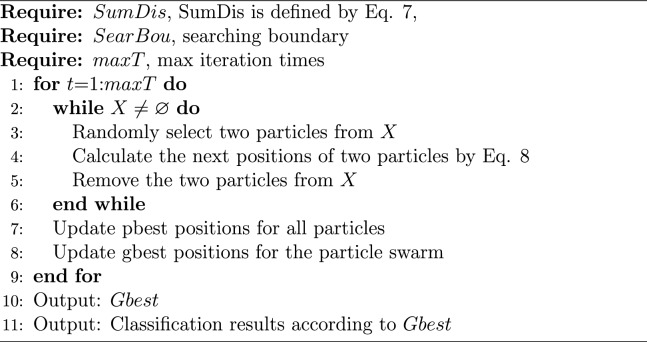
UDLM

### Novel breast cancer image classification model

**Figure 1:**
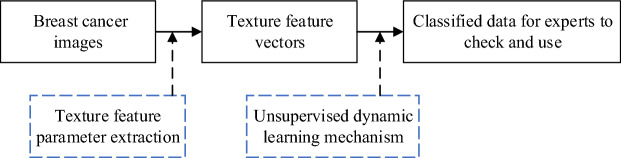
The flowchart of the novel breast cancer image classification model.

The novel breast cancer image classification model (BCICM) is a tool used to reduce the workload of healthcare workers. First, TFPE extracts the texture features of an image and converts the image into a matrix. Then, the UDLM randomly generates a large number of preparatory clustering centers and moves the positions of these preparatory centers by dynamic learning until the iteration reaches the preset upper limit. Finally, the BCICM will output the classification results to the medical staff for their reference and use. To better present this process, the flow chart of the BCICM is shown in Fig. 1. TFPE and UDLM were chosen due to their efficacy in effectively handling multi-scale texture features and utilizing an unsupervised dynamic learning mechanism for classifying these feature vectors. Consequently, the selection of these two methods aims to address the issues inherent in traditional machine learning approaches for medical image classification, which typically require extensive labeled data and prolonged learning periods, thereby mitigating training costs and enhancing applicability. Codes of BCICM can be found at https://github.com/GuoJia-Lab-AI/breast-cancer-image-classification.

## Experiments and results

### Datasets

The BreakHis^14^ database contains microscopic biopsy images of benign and malignant breast tumors. Two well-known traditional methods, random forest (RF) and support vector machine (SVM), were used for the control group.

Benign breast cancers are mainly composed of adenosis, fibroadenoma, phyllodes tumor, and tubular adenoma. Malignant breast cancers are mainly composed of ductal carcinoma, lobular carcinoma in situ, mucinous carcinoma, and papillary carcinoma. All RGB values and corresponding labels were disrupted, and the dataset was split into an 80% training set and a 20% test set using the train test split function.

### Evaluation parameters

The classification of benign and malignant breast tumors is a routine dichotomous task and is usually assessed using accuracy (ACC), sensitivity (SEN), precision (PRE), specificity (SPE), receiver operating characteristic (ROC) curve, and area under the ROC curve (AUC). The model’s classification performance was determined by evaluation indicators such as precision (PRE), specificity (SPE), receiver operating characteristic (ROC) curve, and area under the ROC curve (AUC). Due to the problem of unbalanced data, F1 scores were also used as evaluation metrics to compensate for the negative impact of these metrics on the evaluation of unbalanced data. In this study, a malignant sample is a positive (P) result, and a benign sample is a negative (N) result. A true positive (TP) outcome occurs when the model correctly predicts a malignant sample, a false-negative (FN) outcome occurs when the model incorrectly predicts a malignant sample, a true negative (TN) outcome occurs when the model correctly predicts a benign sample and a false-positive (FP) occurs when the model incorrectly predicts a benign sample. The indicators in the evaluation index can then be expressed. The accuracy (ACC) rate reflects the overall accuracy of the model’s prediction results and is the ratio of the number of samples correctly predicted to the total sample size. The ACC is defined in Eq. (9).

9 $$\begin{aligned} \textrm{ACC}=\frac{(\textrm{TP}+\textrm{TN})}{(P+N)} \times 100 \% \end{aligned}$$

The ratio of the sensitivity (SEN) of malignant tumors, with a higher value indicates that the model can find as many malignant tumors as possible and has a lower underdiagnosis rate. The SEN is defined in Eq. (10).

10 $$\begin{aligned} \textrm{SEN}=\frac{\textrm{TP}}{(\textrm{TP}+\textrm{TN})} \times 100 \% \end{aligned}$$

The precision (PRE) rate reflects the ratio of malignancy samples correctly detected by the model to all predicted malignancy samples. The PRE is defined in Eq. (11).

11 $$\begin{aligned} \textrm{PRE}=\frac{\textrm{TP}}{(\textrm{TP}+\textrm{FP})} \times 100 \% \end{aligned}$$

The specificity (SPE) reflects the ability of the model to detect benign tumors, with higher values indicating a lower rate of misdiagnosis. The SPE is defined in Eq. (12).

12 $$\begin{aligned} \textrm{SPE}=\frac{\textrm{TN}}{(\textrm{TN}+\textrm{FP})} \times 100 \% \end{aligned}$$

The F1 score is a more balanced reflection of the classification performance of the model when the categories are not balanced. The F1 score is defined in Eq. (13).

13 $$\begin{aligned} \textrm{F} 1=\frac{2 \times (\textrm{PRE} \times \textrm{SEN})}{(\textrm{PRE}+\textrm{SEN})} \times 100 \% \end{aligned}$$

### Comparative experimental analysis

#### Effect of different resolution images on feature extraction results

The effect of different resolution images on the feature extraction results is shown in Table 1. As shown in Table 1, there are differences in the texture feature values extracted from different resolutions. Specifically, the energy (ASM) value reflects the uniformity of the grey distribution of the image, and it can be observed that its change is smaller as the resolution increases. The contrast (CON) reflects the clarity of the image texture, and it can be seen that its value is smaller as the resolution increases. The inverse moment difference (IDM) reflects the size of the change in the local texture of the image, and it can be seen that its value decreases as the resolution increases. The entropy (ENT) reflects the amount of information in the image, and it can be found that its change with the resolution is not obvious. From the viewpoint of running time, the higher the resolution is, the longer the processing time, and the model accuracy improvement effect is not obvious.

**Table 1:** Effect of different resolution images on the feature extraction results.

Resolution	GLCM feature values				Running time (ms)	ACC
	ASM	CON	IDM	ENT		
40	0.06105	2.73671	0.59512	3.41055	0.19	0.719
100	0.05758	2.75105	0.60572	3.53628	0.21	0.737
200	0.06644	1.50974	0.68398	3.29359	0.17	0.669
400	0.06636	0.96784	0.73918	3.25522	0.22	0.716

#### Comparison of the results of the different methods

**Figure 2:**
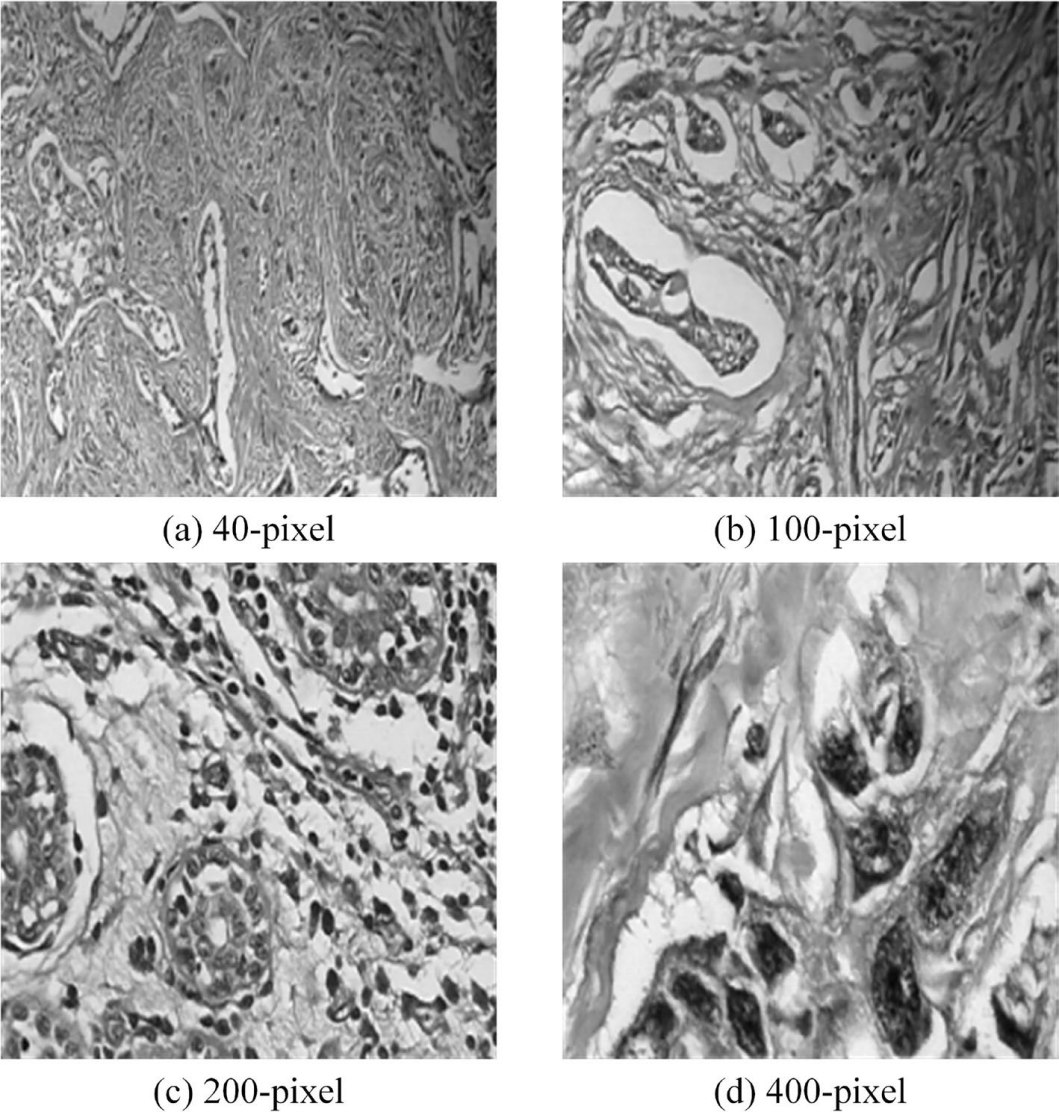
The pre-processing results.

To test the performance of the BCICM in all aspects, 4 sets of simulation tests, including a 40-pixel test, 100-pixel test, 200-pixel test, and 400-pixel test, were implemented. The pre-processing images in the 40-pixel test, 100-pixel test, 200-pixel test, and 400-pixel test are shown in Fig. 2. Selecting images of different pixel sizes for breast cancer image classification experiments serves several important purposes: (1) Multiscale Feature Analysis: Features in breast cancer images may exist at different scales. By using multiple pixel sizes, we can capture image features at various scales, thereby enhancing the performance of the classification model. (2) Adaptation to Different Resolution Requirements: In practical applications, breast cancer images may vary in resolution. Hence, it is essential to test the performance of the classification model on images with different resolutions. Selecting images of different pixel sizes simulates this scenario, enabling the model to generalize better. (3) Exploration of Optimal Performance at Different Sizes: Experimenting with different pixel sizes helps determine which size is most effective for breast cancer image classification tasks. This facilitates the optimization of model design and image processing procedures, thereby improving classification accuracy. (4) Consideration of Computational Costs and Efficiency: Higher-resolution images typically entail greater computational resources and processing time. Selecting appropriate pixel sizes can balance classification accuracy with computational costs and processing time, making the algorithm more practical. Thus, conducting experiments on images with pixel sizes ranging from 40 to 400 pixels enables a comprehensive evaluation of the algorithm’s performance across different resolutions, providing a more holistic solution for breast cancer image classification.

**Figure 3:**
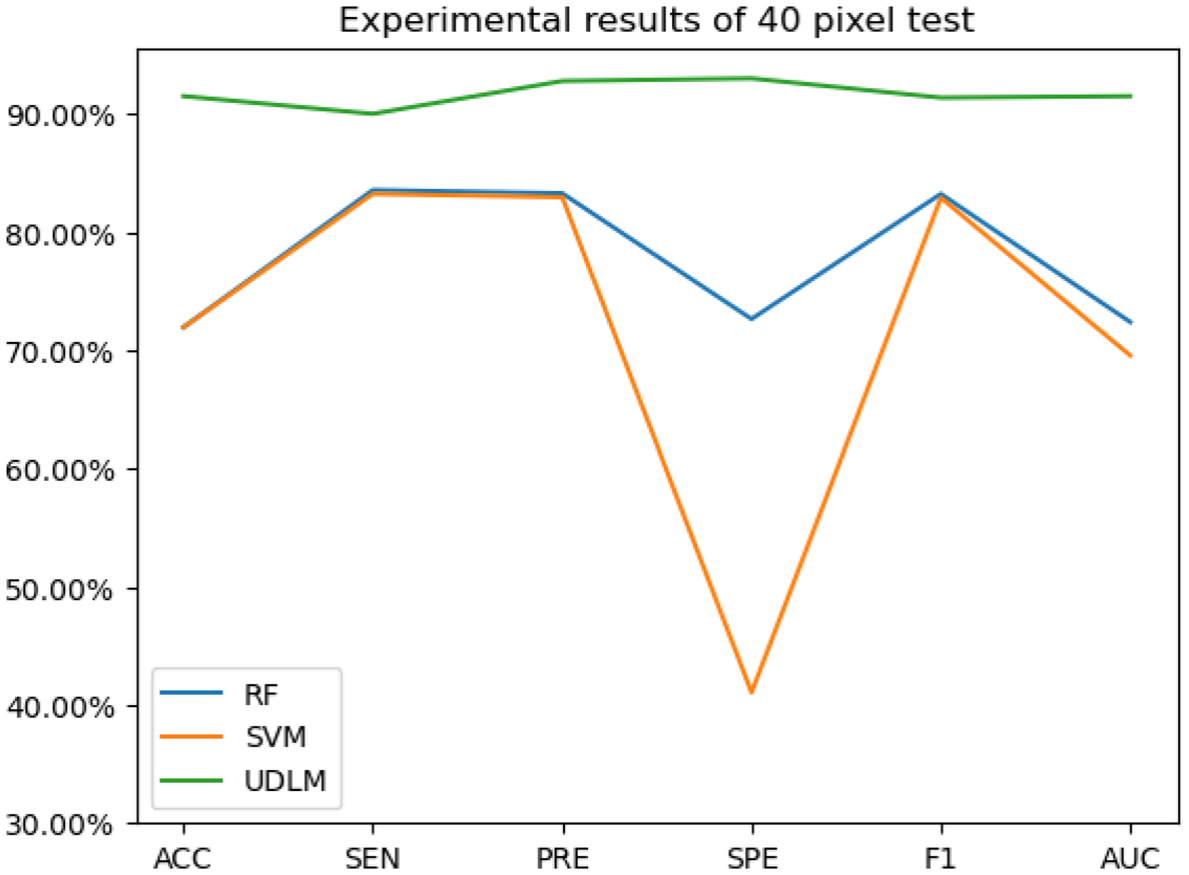
The experimental results of the 40-pixel test.

**Figure 4:**
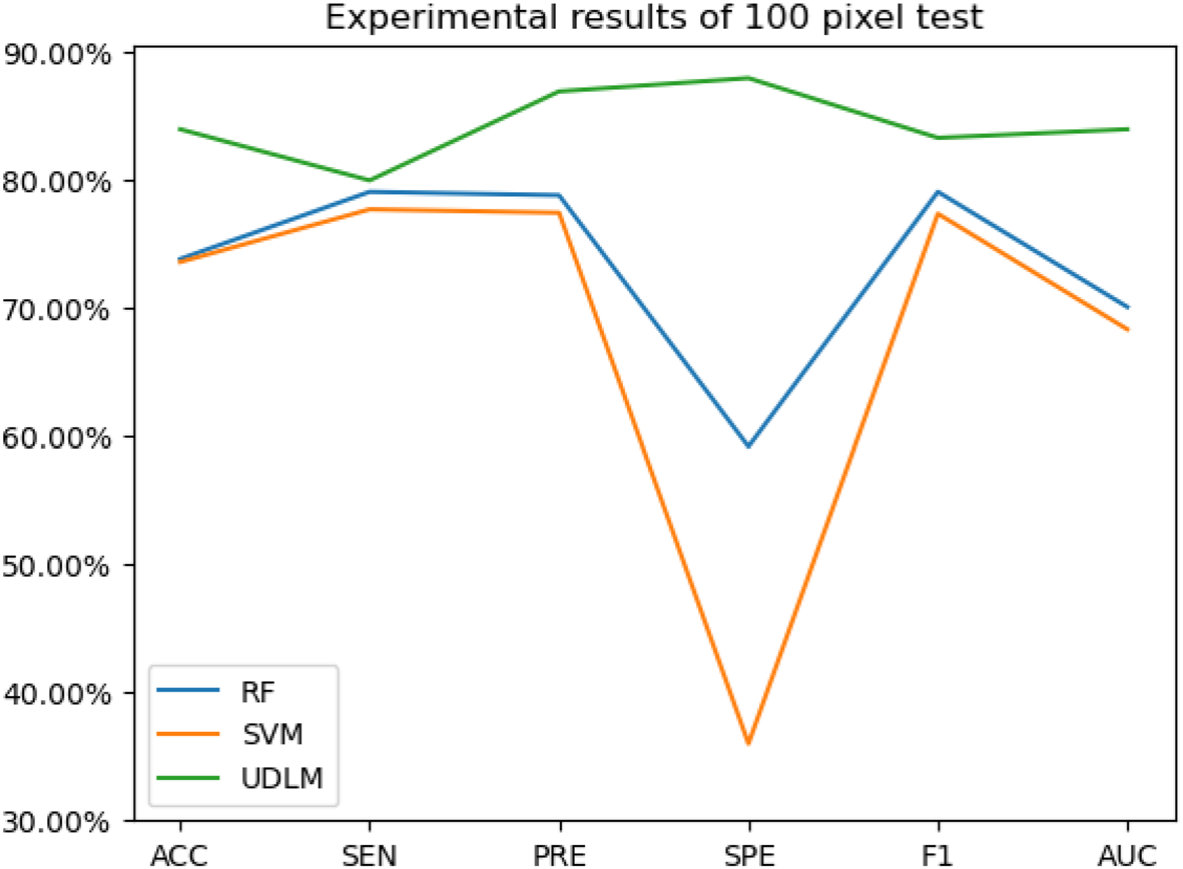
The experimental results of the 100-pixel test.

**Figure 5:**
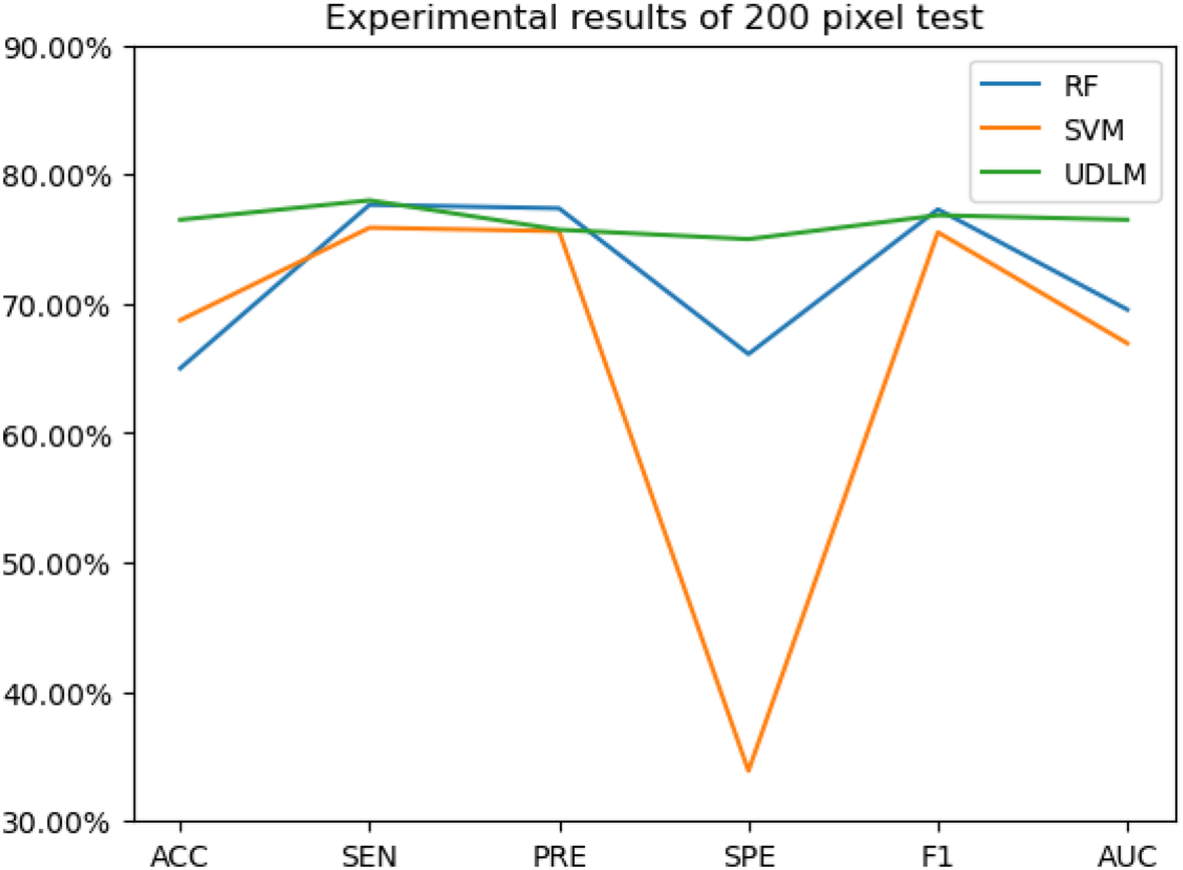
The experimental results of the 200-pixel test.

**Figure 6:**
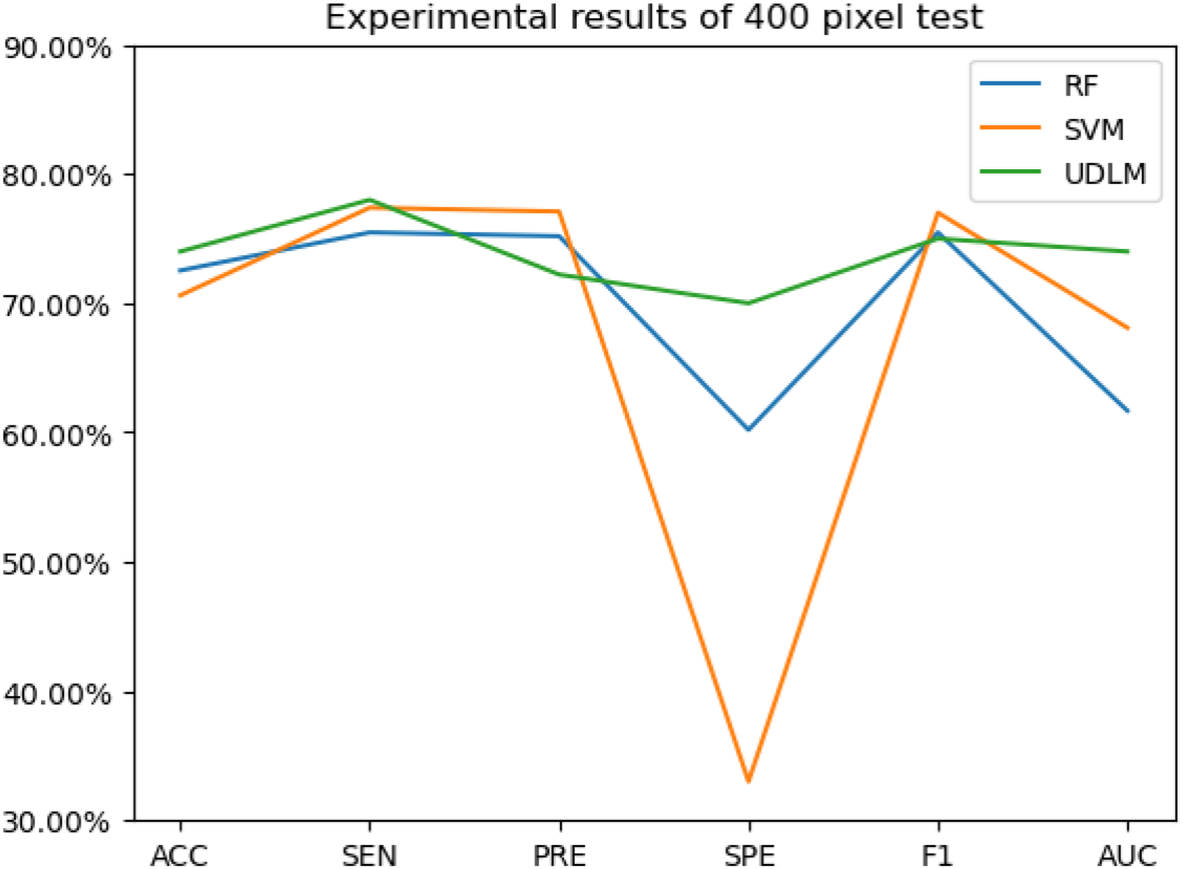
The experimental results of the 400-pixel test.

The experimental results are given in both tables and figures. The results of the 40-pixel test are shown in Table 2 and Fig. 3, the results of the 100-pixel test are shown in Table 3 and Fig. 4, the results of the 200-pixel test are shown in Table 4 and Fig. 5, and the results of the 400-pixel test are shown in Table 5 and Fig. 6. To classify benign and malignant breast tumors, we selected a series of evaluation metrics, such as the SEN, PRE, SPE, F1 score, and callback curve, in addition to the accuracy. Unlike the ACC, which reflects the overall accuracy of the model classification and prediction, the SEN can reflect the leakage rate of the model, and a higher value represents a lower probability of malignant tumors being missed. A higher PRE indicates a higher probability of correctly diagnosing malignant tumors. The SPE represents the ability of the model to detect benign tumors, and a higher value indicates a lower probability of misdiagnosis of benign cases. The F1 score is a combination of the detection rate and specificity of the model, and a higher value indicates a better overall performance of the model. In addition, the AUC value indicates the area under the receiver operating characteristic (ROC) curve, and the closer to 1 the value is, the better the classification effect of the model. It can be seen that the evaluation parameters selected by the method in this paper are optimal compared with those of the benchmark method at all resolutions.

**Table 2:** Experimental results of the 40-pixel test.

Method	ACC (%)	SEN (%)	PRE (%)	SPE (%)	F1 (%)	AUC (%)
RF	71.930	83.553	83.279	72.632	83.224	72.377
SVM	71.930	83.224	82.951	41.053	82.895	69.543
UDLM	91.500	90.000	92.780	93.000	91.370	91.500

**Table 3:** Experimental results of the 100-pixel test.

Method	ACC (%)	SEN (%)	PRE (%)	SPE (%)	F1 (%)	AUC (%)
RF	73.861	79.110	78.840	59.200	79.110	70.105
SVM	73.621	77.740	77.474	36.000	77.397	68.359
UDLM	84.000	80.000	86.960	88.000	83.330	84.000

**Table 4:** Experimental results of the 200-pixel test.

Method	ACC (%)	SEN	PRE	SPE	F1	AUC
RF	65.012	77.660	77.385	66.116	77.305	69.555
SVM	68.735	75.887	75.618	33.884	75.532	66.948
UDLM	76.500	78.000	75.730	75.000	76.850	76.500%

**Table 5:** Experimental results of the 400-pixel test.

Method	ACC	SEN	PRE	SPE	F1	AUC
RF	72.528	75.479	75.191	60.194	75.479	61.678
SVM	70.604	77.395	77.099	33.010	77.011	68.101
UDLM	74.000	78.000	72.220	70.000	75.000	74.000

**Figure 7:**
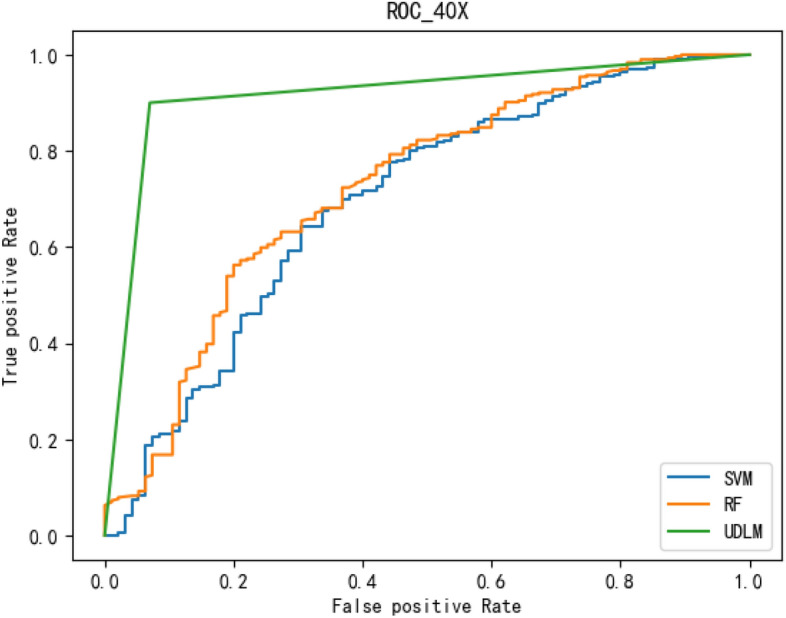
The ROC of the 40-pixel test.

**Figure 8:**
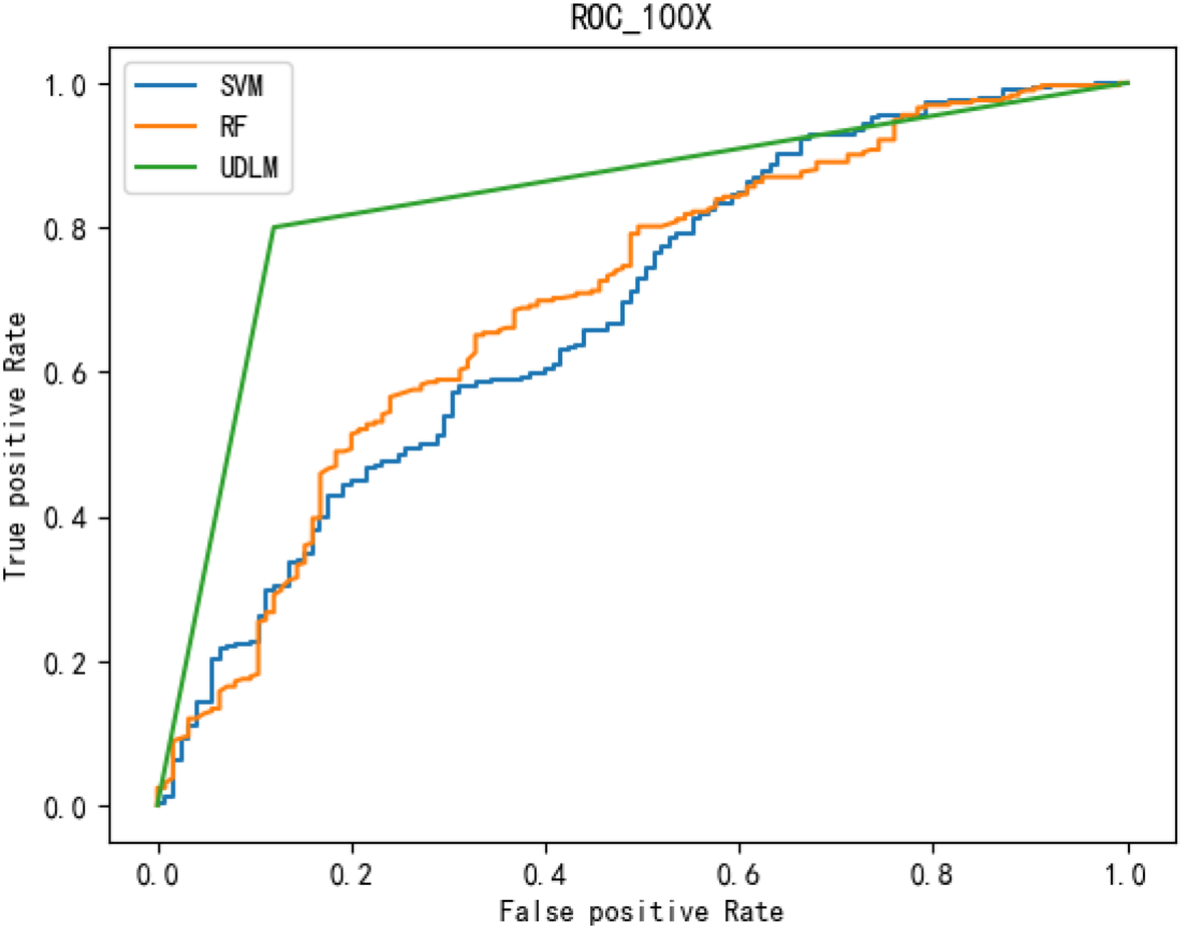
The ROC of the 100-pixel test.

**Figure 9:**
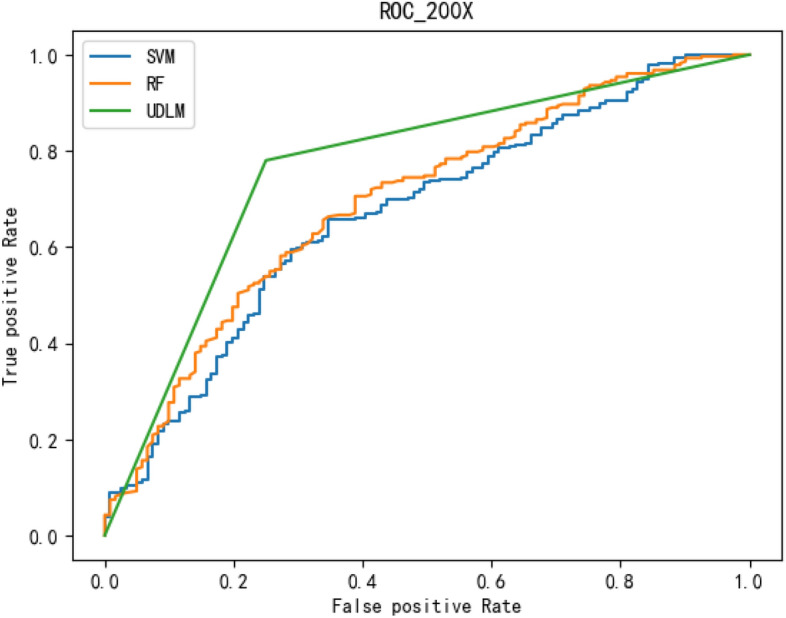
The ROC of the 200-pixel test.

**Figure 10:**
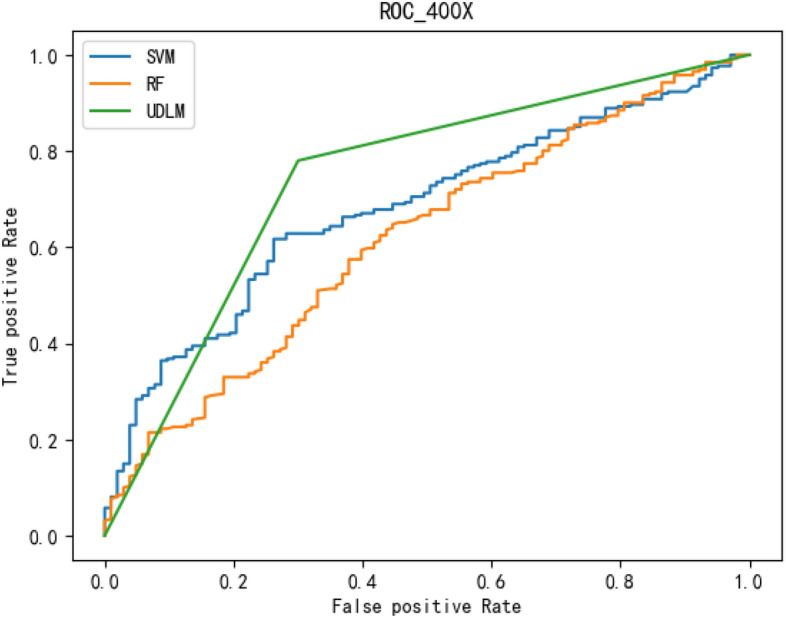
The ROC of the 400-pixel test.

To better demonstrate the performance of the BCICM, we plotted the receiver operating characteristic (ROC) curve for four sets of experiments. The curves clearly demonstrate that the proposed algorithm has a great advantage in all experiments. The ROC curve of the 40-pixel test is shown in Fig. 7. The ROC curve of the 100-pixel test is shown in Fig. 8. The ROC curve of the 200-pixel test is shown in Fig. 9. The ROC curve of the 400-pixel test is shown in Fig. 10.

From the above results, it can be seen that the proposed model is more sensitive to the tumor region during classification. However, it responds to some information in the background to a lesser extent. This shows that the model can learn richer information about the tumor region. The feedback in the calcified region and nonsmooth edges of the tumor is also more obvious in the malignant tumor images.

However, as the image resolution increases, the main reason for the decrease in model accuracy may be attributed to the fact that high-resolution images contain more details and noise, posing more complex challenges for the model during processing. High-resolution images may encompass finer structures and textures, rendering it difficult for the model to accurately differentiate and classify various features. Moreover, high-resolution images may increase the dimensionality of the feature space, making the model more prone to overfitting or encountering greater computational complexity during training.

Addressing this issue, future work could explore the following avenues: Firstly, designing and employing more effective feature extraction methods tailored to high-resolution images to extract features that are most discriminative and robust for the classification task. Secondly, researching and optimizing model structures to better adapt to and handle the characteristics of high-resolution images, including increasing model capacity or introducing more complex network architectures. Additionally, exploring the use of data augmentation techniques to expand the training dataset to alleviate overfitting issues associated with high-resolution images. Lastly, approaches such as transfer learning or semi-supervised learning to leverage pre-trained models or a small amount of labeled data to enhance the model’s generalization ability on high-resolution images.

In conclusion, addressing the decrease in model accuracy with increasing image resolution requires future work to focus on improving feature extraction methods, optimizing model structures, expanding datasets, and employing strategies such as transfer learning, aiming to enhance model performance and generalization ability on high-resolution images.

## Conclusion

This work introduces a novel breast cancer image classification model (BCICM) that achieves unsupervised classification of breast cancer images through the collaboration between TFPE and the UDLM. Across all four different size tests, the BCICM demonstrates the highest accuracy results. These experimental findings underscore the BCICM’s capacity to offer efficient and highly accurate support to medical professionals.

Given the unsupervised nature of the BCICM, it holds theoretical applicability to various types of image classification tasks. Therefore, leveraging the BCICM for the classification of other cancer images, such as lung cancer images and skin cancer images, appears to be a viable future direction. Moreover, there exists room for improvement in the adaptive evolution strategy of the BCICM. Hence, our future endeavors aim to propose enhanced dynamic learning methods to enhance the algorithm’s efficiency and accuracy.

Furthermore, it is observed that the accuracy of the BCICM diminishes as the image resolution increases. Thus, we emphasize the importance of enhancing the classification accuracy of the BCICM in high-resolution images for future investigations. Additionally, augmenting the diversity of datasets and elucidating their fidelity to real-world contexts are deemed crucial for bolstering the resilience of research outcomes. This avenue represents a critical aspect for future inquiry.

## Data Availability

The datasets used and/or analyzed during the current study are available from the corresponding author upon reasonable request.

## References

[1] Zhu C, Song F, Wang Y, Dong H, Guo Y, Liu J (2019). Breast cancer histopathology image classification through assembling multiple compact CNNs. BMC Med. Inform. Decis. Mak..

[2] Zhu C, Chen W, Peng T, Wang Y, Jin M (2022). Hard sample aware noise robust learning for histopathology image classification. IEEE Trans. Med. Imaging.

[3] Wang, S. *et al.**Artificial Intelligence in Lung Cancer Pathology Image Analysis*10.3390/cancers11111673 (2019). 10.3390/cancers11111673 PMC6895901 31661863

[4] Yokoya, N., Chan, J.C., Segl, K. Potential of Resolution-Enhanced Hyperspectral Data for Mineral Mapping Using Simulated EnMAP and Sentinel-2 Images. 10.3390/rs8030172 (2016).

[5] Qing Y, Liu W, Feng L, Gao W (2021). Improved transformer net for hyperspectral image classification. Remote Sens..

[6] Chen Y, Jiang H, Li C, Jia X, Ghamisi P (2016). Deep feature extraction and classification of hyperspectral images based on convolutional neural networks. IEEE Trans. Geosci. Remote Sens..

[7] Song B, Li S, Sunny S, Gurushanth K, Mendonca P, Mukhia N, Patrick S, Gurudath S, Raghavan S, Tsusennaro I, Leivon ST, Kolur T, Shetty V, Bushan V, Ramesh R, Peterson T, Pillai V, Wilder-Smith P, Sigamani A, Suresh A, Kuriakose MA, Birur P, Liang R (2021). Classification of imbalanced oral cancer image data from high-risk population. J. Biomed. Opt..

[8] Khairi SSM, Bakar MAA, Alias MA, Bakar SA, Liong CY, Rosli N, Farid M (2022). Deep learning on histopathology images for breast cancer classification: a bibliometric analysis. Healthcare.

[9] Bhavya Sai V, Narasimha Rao G, Ramya M, Sujana Sree Y, Anuradha T (2018). Classification of skin cancer images using tensorflow and inception V3. Int. J. Eng. Technol..

[10] Sharma R, Singh A, Kavita, Jhanjhi NZ, Masud M, Sami Jaha E, Verma S (2022). Plant disease diagnosis and image classification using deep learning. Comput. Mater. Continua.

[11] Wang X, Chen H, Gan C, Lin H, Dou Q, Tsougenis E, Huang Q, Cai M, Heng PA (2020). Weakly supervised deep learning for whole slide lung cancer image analysis. IEEE Trans. Cybern..

[12] Li X, Jiao H, Wang Y (2020). Edge detection algorithm of cancer image based on deep learning. Bioengineered.

[13] Sudharshan PJ, Petitjean C, Spanhol F, Oliveira LE, Heutte L, Honeine P (2019). Multiple instance learning for histopathological breast cancer image classification. Expert Syst. Appl..

[14] Spanhol FA, Oliveira LS, Petitjean C, Heutte L (2016). A dataset for breast cancer histopathological image classification. IEEE Trans. Biomed. Eng..

[15] Mou L, Ghamisi P, Zhu XX (2017). Deep recurrent neural networks for hyperspectral image classification. IEEE Trans. Geosci. Remote Sens..

[16] Hu W, Huang Y, Wei L, Zhang F, Li H (2015). Deep convolutional neural networks for hyperspectral image classification. J. Sens..

[17] Song W, Li S, Fang L, Lu T (2018). Hyperspectral image classification with deep feature fusion network. IEEE Trans. Geosci. Remote Sens..

[18] Beeravolu AR, Azam S, Jonkman M, Shanmugam B, Kannoorpatti K, Anwar A (2021). Preprocessing of breast cancer images to create datasets for deep-CNN. IEEE Access.

[19] Xu Y, Zhu JY, Chang EI, Lai M, Tu Z (2014). Weakly supervised histopathology cancer image segmentation and classification. Med. Image Anal..

[20] Zhong Z, Li J, Luo Z, Chapman M (2018). Spectral-spatial residual network for hyperspectral image classification: A 3-D deep learning framework. IEEE Trans. Geosci. Remote Sens..

[21] Zhu L, Chen Y, Ghamisi P, Benediktsson JA (2018). Generative adversarial networks for hyperspectral image classification. IEEE Trans. Geosci. Remote Sens..

[22] Mei S, Ji J, Geng Y, Zhang Z, Li X, Du Q (2019). Unsupervised spatial-spectral feature learning by 3D convolutional autoencoder for hyperspectral classification. IEEE Trans. Geosci. Remote Sens..

[23] Li X, Radulovic M, Kanjer K, Plataniotis KN (2019). Discriminative pattern mining for breast cancer histopathology image classification via fully convolutional autoencoder. IEEE Access.

[24] Wang X, Tan K, Du Q, Chen Y, Du P (2019). Caps-TripleGAN: GAN-assisted CapsNet for hyperspectral image classification. IEEE Trans. Geosci. Remote Sens..

[25] Hameed Z, Zahia S, Garcia-Zapirain B, Aguirre JJ, Vanegas AM (2020). Breast cancer histopathology image classification using an ensemble of deep learning models. Sensors.

[26] Yan R, Ren F, Wang Z, Wang L, Zhang T, Liu Y, Rao X, Zheng C, Zhang F (2020). Breast cancer histopathological image classification using a hybrid deep neural network. Methods.

[27] Feng Y, Zhang L, Mo J (2020). Deep manifold preserving autoencoder for classifying breast cancer histopathological images. IEEE/ACM Trans. Comput. Biol. Bioinform..

[28] Nguyen, H. D., Vu, X. S. & Le, D. T. Modular graph transformer networks for multi-label image classification. In *35th AAAI Conference on Artificial Intelligence, AAAI 2021*, Vol. 10B. 10.1609/aaai.v35i10.17098 (2021).

[29] Hu X, Li T, Zhou T, Liu Y, Peng Y (2021). Contrastive learning based on transformer for hyperspectral image classification. Appl. Sci..

[30] Boumaraf S, Liu X, Wan Y, Zheng Z, Ferkous C, Ma X, Li Z, Bardou D (2021). Conventional machine learning versus deep learning for magnification dependent histopathological breast cancer image classification: A comparative study with visual explanation. Diagnostics.

[31] Dai Y, Gao Y, Liu F (2021). Transmed: Transformers advance multi-modal medical image classification. Diagnostics.

[32] Wang P (2021). Automatic classification of breast cancer histopathological images based on deep feature fusion and enhanced routing. Biomed. Signal Process. Control.

[33] Thilagaraj M, Arunkumar N, Govindan P (2022). Classification of breast cancer images by implementing improved DCNN with artificial fish school model. Comput. Intell. Neurosci..

[34] Hong D (2022). SpectralFormer: Rethinking hyperspectral image classification with transformers. IEEE Trans. Geosci. Remote Sens..

[35] Liu Y, Dou Y, Jin R, Li R, Qiao P (2022). Hierarchical learning with backtracking algorithm based on the Visual Confusion Label Tree for large-scale image classification. Vis. Comput..

[36] Guo J, Zhou G, Yan K, Sato Y, Di Y (2023). Pair barracuda swarm optimization algorithm: A natural-inspired metaheuristic method for high dimensional optimization problems. Sci. Rep..

[37] Guo J, Zhou G, Yan K, Shi B, Di Y, Sato Y (2023). A novel hermit crab optimization algorithm. Sci. Rep..

[38] Guo J, Zhou G, Di Y, Shi B, Yan K, Sato Y (2023). A bare-bones particle swarm optimization with crossed memory for global optimization. IEEE Access.

